# Microparticle-Induced Coagulation Relates to Coronary Artery Atherosclerosis in Severe Aortic Valve Stenosis

**DOI:** 10.1371/journal.pone.0151499

**Published:** 2016-03-24

**Authors:** Patrick Horn, Gülsüm Erkilet, Verena Veulemans, Patric Kröpil, Leon Schurgers, Tobias Zeus, Christian Heiss, Malte Kelm, Ralf Westenfeld

**Affiliations:** 1 Division of Cardiology, Pulmonology, and Vascular Medicine, Medical Faculty, University Düsseldorf, Düsseldorf, Germany; 2 Department of Diagnostic and Interventional Radiology, Medical Faculty, University Düsseldorf, Düsseldorf, Germany; 3 Department of Biochemistry, Cardiovascular Research Institute Maastricht (CARIM), Maastricht University, Maastricht, The Netherlands; 4 Cardiovascular Research Institute Düsseldorf (CARID), Medical Faculty, University Düsseldorf, Düsseldorf, Germany; KRH Robert Koch Klinikum Gehrden, GERMANY

## Abstract

**Background:**

Circulating microparticles (MPs) derived from endothelial cells and blood cells bear procoagulant activity and promote thrombin generation. Thrombin exerts proinflammatory effects mediating the progression of atherosclerosis. Aortic valve stenosis may represent an atherosclerosis-like process involving both the aortic valve and the vascular system. The aim of this study was to investigate whether MP-induced thrombin generation is related to coronary atherosclerosis and aortic valve calcification.

**Methods:**

In a cross-sectional study of 55 patients with severe aortic valve stenosis, we assessed the coronary calcification score (CAC) as indicator of total coronary atherosclerosis burden, and aortic valve calcification (AVC) by computed tomography. Thrombin-antithrombin complex (TATc) levels were measured as a marker for thrombin formation. Circulating MPs were characterized by flow cytometry according to the expression of established surface antigens and by measuring MP-induced thrombin generation.

**Results:**

Patients with CAC score below the median were classified as patients with *low* CAC, patients with CAC Score above the median as *high* CAC. In patients with *high* CAC compared to patients with *low* CAC we detected higher levels of TATc, platelet-derived MPs (PMPs), endothelial-derived MPs (EMPs) and MP-induced thrombin generation. Increased level of PMPs and MP-induced thrombin generation were independent predictors for the severity of CAC. In contrast, AVC Score did not differ between patients with *high* and *low* CAC and did neither correlate with MPs levels nor with MP-induced thrombin generation.

**Conclusion:**

In patients with severe aortic valve stenosis MP-induced thrombin generation was independently associated with the severity of CAC but not AVC indicating different pathomechanisms involved in coronary artery and aortic valve calcification.

## Introduction

Atherosclerosis is a chronic inflammatory disease characterized by endothelial dysfunction, local inflammation, leukocyte transmigration, and binding of monocytes to the arterial vessel wall [1]. Aortic valve stenosis is independently associated with cardiovascular risk factors and clinically apparent cardiovascular disease, thus some authors claim that the degeneration of the aortic valve could represent an atherosclerosis-like process involving both the aortic valve as well as the vascular system [2, 3]. Apoptosis, inflammatory activation and cellular stress occurring during atherosclerosis development induce the formation of microparticles (MPs) [4, 5]. MPs are shed membrane particles of less than a micrometer in diameter thought to be budded into the circulation from endothelial cells and various blood cells, including platelets, leukocytes and erythrocytes. MPs have been established as biomarkers that predict adverse cardiovascular outcome [6–8]. In patients with atherosclerotic diseases such as coronary artery disease (CAD) as well as in patients with severe aortic valve stenosis level of circulating MPs are increased within the vascular compartment as compared to healthy subjects [4, 9, 10].

A few studies demonstrated an association between level of MPs and the *severity* of atherosclerotic processes in diabetic patients and in postmenopausal woman [11, 12]. It is not known, whether the level of circulating MPs are associated with the severity of aortic valve calcification and with the severity of coronary atherosclerosis in patients with advanced calcification.

Furthermore, it is poorly understood whether MPs are only a result of atherosclerotic modifications affecting the vascular compartment or also play a role in the pathogenesis of atherosclerosis progression. MPs possess procoagulant activity that relies mainly on the expression of phosphatidylserine and tissue factor promoting the generation of plasma thrombin [13, 14]. Thrombin is not only the central protease of the coagulation cascade but also acts as a strong proinflammatory mediator with effects on endothelial cells, vascular smooth muscle cells, monocytes, and platelets, all of which are involved in the pathophysiology of atherosclerosis progression and vascular calcification [15, 16]. Enhanced plasma thrombin generation predicts the presence and severity of coronary artery calcification (CAC) [17]. In patients with severe aortic valve stenosis plasma thrombin generation is increased potentially due to the hemostatic effect of turbulent flow through the stenosis [18]. Taken together, the association between level of MPs, MP-induced thrombin generation, and the severity of coronary and valvular calcification is not known.

The aim of this study was to investigate in patients with severe aortic valve stenosis and advanced calcification whether level of MPs and MP-induced thrombin generation are related to coronary atherosclerosis as assessed by CAC score and related to aortic valve calcification as assessed by aortic valve calcification (AVC) score.

## Materials and Methods

### Study design

We analyzed patients CT data and MPs in frozen plasma samples that were stored during our previously published clinical study showing in patients with severe aortic valve stenosis that endothelial function improved and level of circulating MPs decreased following transcatheter aortic valve implantation (TAVI) [10]. In this cross-sectional study a total of 61 patients with symptomatic severe aortic valve stenosis were included who were referred for TAVI. 6 patients were excluded from data analysis as no CT was performed and CAC Score could not be assessed. In 55 patients, we analyzed CAC Score, AVC Score, endothelial function, plasma levels of circulating MPs and MP-induced thrombin generation pre TAVI and assessed transvalvular pressure gradients, peak velocity and valve area by transthoracic echocardiography and cardiac catheterization, respectively.

General exclusion criteria were recent or active inflammation (clinical apparent infection, elevated CRP), stage 5 chronic kidney disease, active malignancies within the last year. This study was conducted according to the guidelines laid down in the Declaration of Helsinki and all procedures involving human subjects and patients were approved by the University of Düsseldorf Committee on Human Research (Clinicaltrials.gov: NCT01993485). Written informed consent was witnessed and formally recorded.

### Coronary artery and aortic valve calcification

Cardiac-MDCT imaging was used according to the standardized recommendations for computer tomography (CT) image acquisition before TAVI procedure in our center. CT data were obtained using a 128 slice single source CT-scanner (“SOMATOM Definition AS+”, Siemens Healthcare, Forchheim, Germany) for state-of-the-art cardiac imaging with high temporal resolution of 150 ms and a collimation of 128×0.6 mm. A non-enhanced scan was performed for coronary and valvular calcium scoring with a tube voltage of 100–120 kV, a pitch of 0.2 and a gantry rotation time of 0.3 s. Axial images were reconstructed with a slice thickness of 3.0 mm. All coronary segments and the aortic valves were analysed separately regarding calcified lesions with the use of software (Syngo Calcium Scoring^®^, Siemens Healthcare, Germany). Data were transferred and stored in the institutional Picture Archiving and Communication System (PACS) (IDS 7 version 15, Sectra, Linköping, Sweden).

### Endothelial function

Endothelial function was assessed by measuring FMD of the brachial artery using ultrasound (Vivid I, GE, Munich, Germany). Baseline data for diameter and mean blood flow velocity of the brachial artery were quantified after 20 min of supine rest following the identical protocol. The image and flow analyses were performed offline from recorded loops with an automated system (Brachial Analyzer 5, Medical Imaging Applications, Coralville, USA). All diameter readings were taken at diastole, and flow velocity represents the mean angle-corrected Doppler flow velocity. Vasodilation results are presented as percent change: (Diameter_postischemia_—Diameter_baseline_/Diameter_baseline_) x 100. Assessment of endothelium independent vasodilatation with nitroglycerine was not performed due to safety concerns regarding the risk for severe hypotension in patients with critical aortic valve stenosis.

### Circulating MPs as marker for endothelial integrity

MP subpopulations were discriminated by flow cytometry according to the expression of established surface antigens as described previously [19]. All biological samples were stored and processed in an identical way. Briefly, citrated blood (6 ml) was drawn from the cubital vein. Platelet-free plasma (PFP) was obtained immediately by successive centrifugations of the supernatants at 300*g* and 10,000*g* for 5 min at RT. PFP obtained by this sequential centrifugation contains MPs but no platelets, as shown by flow cytometry and fluorescence-based laser-scanning microscopy. PFP were stored directly at -80°C. FACS analysis was performed upon 3 months following the identical protocol. PFP were incubated for 30 min with fluorochrome-labeled antibodies or matching isotype controls and analyzed in a Canto II flow cytometer (Beckton Dickinson, Heidelberg, Germany) (S1 Fig). MPs were distinguished from smaller exosomes (0.04–0.1 μm), which originate from the endoplasmic membranes, and from larger phospholipid vesicles, the apoptotic bodies (>1.0 μm), which contain nuclear material. The microbead standard of 1.0 μm (Polyscience Inc., Eppelheim, Germany) was used as upper limit. The threshold of 0.2 μm was set due to technical inability of the flow cytometer to detect particles <0.2 μm [20]. Thus MPs ranging from 0.1–0.2 μm were not accounted for. EMP subpopulations were defined as CD144^+^, CD62E^+^ or CD31^+^/CD41^-^. Platelet-derived MPs (PMPs) were defined as CD41^+^-MPs. The total number of MPs was quantified with flow count calibrator beads (Beckman Coulter; 20 μl). Unless specified otherwise, chemicals were purchased from Sigma Aldrich (Deisenhofen, Germany).

### Thrombin generating capacity of plasma—thrombin/ antithrombin III complex (TATc)

TATc level as a marker for thrombin generation in plasma were measured in platelet rich plasma using commercial microenzyme immunoassay kit (Enzygnost TAT Micro, Dade Behring, Marburg, Germany).

### Thrombin generating capacity of MPs

MP procoagulative activity was assessed using a two-step amidolytic assay (Zymuphen MP-Activity kit, Hyphen BioMed, Neuville-sur-Oise, France). The diluted PFP, supplemented with calcium, Factor Xa and thrombin inhibitors, was introduced into one of the microplate wells coated with Streptavidine and biotinylated Annexin V. After incubation FV, FX and prothrombin were introduced to form the prothrombinase complex FXa-FVa. When presented in the tested sample, MPs bound to Annexin V and exposed their phospholipids surface allowing the prothrombinase complex, in presence of calcium, to cleave prothrombin into thrombin. The kinetic of the reaction and thrombin generation was assessed by measuring its specific activity on the thrombin substrate at different time points (0–10 min) as indicated compared to the reaction with calibrator samples. The reaction was stopped with 2% Citric Acid and absorbance was measured at 405 nm. As control, we used MP-depleted plasma (supernatant obtained by ultracentrifugation of the PFP at 30,000*g* for 90 min at 4°C) not able to trigger thrombin generation significantly in this assay.

### Statistics

Continuous data are presented as mean ± standard deviation of the mean (SD), categorical data as absolute numbers. Patient characteristics were compared using unpaired t test (continuous data) and 2-tailed Fisher’s exact test (categorical data). The D'Agostino & Pearson omnibus normality test was used to confirm normal distribution of hemodynamic parameters, FMD values, TAT Complex and Calcification Scores and non-normal distribution of MP levels.

Patients with CAC Score below the median (Agatston Score 974) were compared with patients with CAC Score above the median using unpaired t test (FMD, thrombin generation) or Mann Whitney U Test (non-normally distributed data).

Correlation of hemodynamic parameters was assessed by Pearson coefficient, correlation of MPs was assessed by Spearman coefficient. P-values <0.05 were considered as statistically significant. Multivariate regression model was used to determine the influence of multiple parameters on CAC. Variables for the linear regression model were chosen based on simple correlation analysis and those variables known or thought to be associated with CAC. Only variables with p<0.05 in univariate analysis were included in multivariate analysis. All statistical tests were conducted using SPSS 21.0 (IBM, Armonk, NY, USA) and Prism 5.0 (GraphPad Software, San Diego, CA, USA).

## Results

### Baseline characteristics

In patients with severe aortic valve stenosis mean CAC Score was 1797±2064 and mean AVC Score 4566±4729. Patients with CAC Score below the median of 974 were classified as patients with *low* CAC (437±290, n = 28), patients with CAC Score above the median as *high* CAC (3208±2170, n = 27) (Table 1). The patients were separated based on the median as patients with *high* CAC presented with a higher burden of coronary artery disease: number of coronary vessels affected by coronary stenosis, calculated Syntax Score (17±19 vs. 4±9, p = 0.005) and EuroScore (24±12 vs. 19±8, p = 0.024) were higher in patients with *high* CAC (Table 1) compared to patients with *low* CAC. The groups did not differ between medications and laboratory parameters (Tables 1 and 2).

**Table 1:** Patients‘ characteristics.

*Patients‘ characteristics*	All patients	Low CAC	High CAC	P value
*N (male/female)*	55 (25/30)	28 (11/17)	27 (14/13)	0.140
*Coronary artery parameters*				
*CAC Score*	1797±2064	437±290	3208±2170	<0.001
*Coronary vessels with stenosis (n)*	2±1	1±1	2±1	<0.001
*Syntax Score*	11±16	4±9	17±19	0.005
*Aortic valve parameters*				
*AVC Score*	4385±3838	4353±3755	4417±3994	0.9518
*dPmean (mmHg)*	40±16	37±13	43±18	0.170
*dPmax (mmHg)*	69±26	65±24	74±28	0.293
*Vmax (m/sec)*	4.2±1.1	3.9±0.9	4.4±1.3	0.074
*Age (yrs)*	81±5	82±5	81±6	0.470
*EURO Score (log I)*	21±10	19±8	24±12	0.024
*Body mass index (kg/m*^*2*^*)*	26.6±4.9	27±5.9	26.2±3.7	0.577
*Diabetes (%)*	27%	29%	26%	0.232
*Hypertension (%)*	91%	93%	89%	0.318
*Hyperlipidemia (%)*	87%	82%	92%	0.170
*Prior Smoking (%)*	27%	25%	29%	0.221
*PAD (%)*	33%	29%	37%	0.182
*Carotid artery disease (%)*	24%	18%	30%	0.150
*Medication*				
*Coumarin (%)*	12%	7%	4%	0.389
*Aspirin (%)*	93%	93%	93%	0.389
*Clopidogrel (%)*	65%	67%	63%	0.208
*Renin-Angiotensin System Blocker (%)*	60%	64%	56%	0.175
*Beta Blocker (%)*	82%	86%	78%	0.207
*Statins (%)*	89%	88%	90%	0.331

**Table 2:** Physiological Parameters.

*Physiological parameters*	All patients	Low CAC	High CAC	p value
*Heart Rate (/min)*	72±15	71±15	73±15	0.628
*Systolic Arterial Pressure (mmHg)*	142±17	142±17	143±18	0.847
*Diastolic Arterial Pressure*	68±12	70±14	67±10	0.280
*Laboratory parameters*				
*GFR MDRD formula (ml/min)*	58±17	57±16	58±18	0.857
*Glucose (mg/dl)*	144±36	141±36	146±36	0.354
*Cholesterol (mg/dl)*	175±39	179±36	170±42	0.462
*High-density lipoprotein (mg/dl)*	57±17	60±16	54±19	0.468
*Low-density lipoprotein (mg/dl)*	137±34	134±26	140±41	0.714
*CRP (mg/dl)*	0.6±0.4	0.6±0.4	0.7±0.4	0.825
*White blood cells (1000 per µl)*	7.9±2.7	7.6±3.2	8.2±1.9	0.363
*Erythrocytes (1x10*^*6*^ *per µl)*	4.2±0.6	4.0±0.5	4.3±0.7	0.160
*Platelets (x1000 per µl)*	214±68	224±79	204±52	0.264

### Levels of MPs, endothelial function, and the severity of CAC

**Fig 1:**
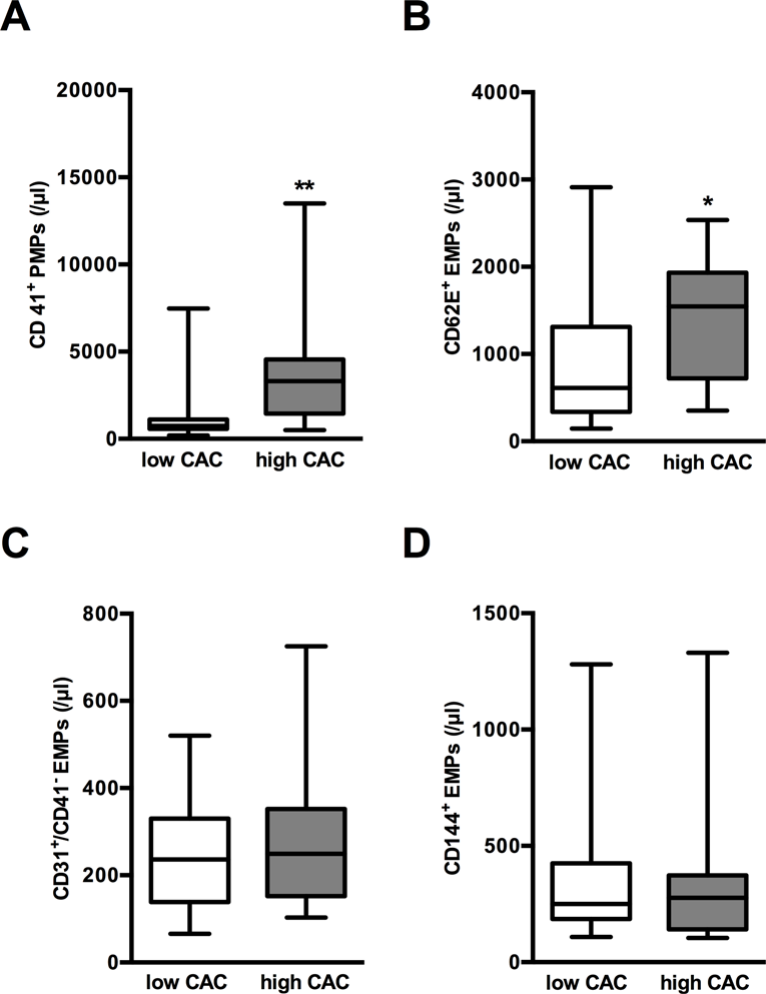
Increased levels of MPs in patients with high CAC. (A) Level of PMPs (CD41^+^) and (B) CD62E^+^ EMPs, but not (C) CD31^+^/Cd41^-^ EMPs and (D) CD144^+^ EMPs were higher in patients with *high* CAC compared to patients with *low* CAC. Data are presented as minimum and maximum (crosses), interquartile range from 25 to 75% (box), mean (square), and median (line) in a box plot. * indicates significant difference of the mean.

In patients with *high* CAC, levels of CD41^+^ PMPs and CD62E^+^ EMPs were greater compared to patients with *low* CAC (4802±4922 /μl vs. 1413±1887 /μl; p<0.001 and 1375±777 /μl vs. 905±784 /μl; p = 0.010) (Fig 1). Levels of CD31^+^/CD41^-^ EMPs and CD144^+^ EMPs did not differ between patients with *high* CAC and *low* CAC (CD31^+^/CD41^-^ EMPs: 265±139 /μl vs. 242±120 /μl, p = 0.6251; CD144^+^ EMPs: 323±271 /μl vs. 371±298 /μl, p = 0.464). There was no difference in FMD as a marker of early endothelial dysfunction between the patient groups (3.0±0.6% vs. 2.9±0.5%; p = 0.597). Level of PMPs (r = 0.698, p<0.001) and CD62E^+^ EMPs (r = 0.439, p = 0.002) but not CD31^+^/CD41^-^ EMPs and CD144^+^ EMPs correlated with the CAC Score (Table 3).

**Table 3:** Univariate Correlation analysis for the association between CAC Score and AVC Score with endothelial function (FMD) and circulating MPs.

	CAC Score	
	r	p Value
Coronary vessel disease (n)	0.560	<0.001
Syntax Score	0.499	<0.001
AVC Score	0.127	0.357
FMD	-0.052	0.709
CD41^+^ PMPs	0.698	<0.001
CD62E^+^ EMPs	0.438	0.001
CD31^+^/CD41^-^ EMPs	-0.016	0.920
CD144^+^ EMPs	-0.090	0.515
MP-induced thrombin generation	0.587	0.001
TAT complex	0.269	0.047
	AVC Score	
	r	p Value
Coronary Vessel disease (n)	-0.041	0.789
Syntax Score	-0.139	0.335
FMD	-0.064	0.668
CD41^+^ PMPs	0.053	0.698
CD62E^+^ EMPs	0.208	0.578
CD31^+^/CD41^-^ EMPs	0.085	0.533
CD144^+^ EMPs	-0.174	0.203
Thrombin generation	0.208	0.128
TAT complex	0.006	0.968

### TATc levels, MP-induced thrombin generation and the severity of CAC

**Fig 2:**
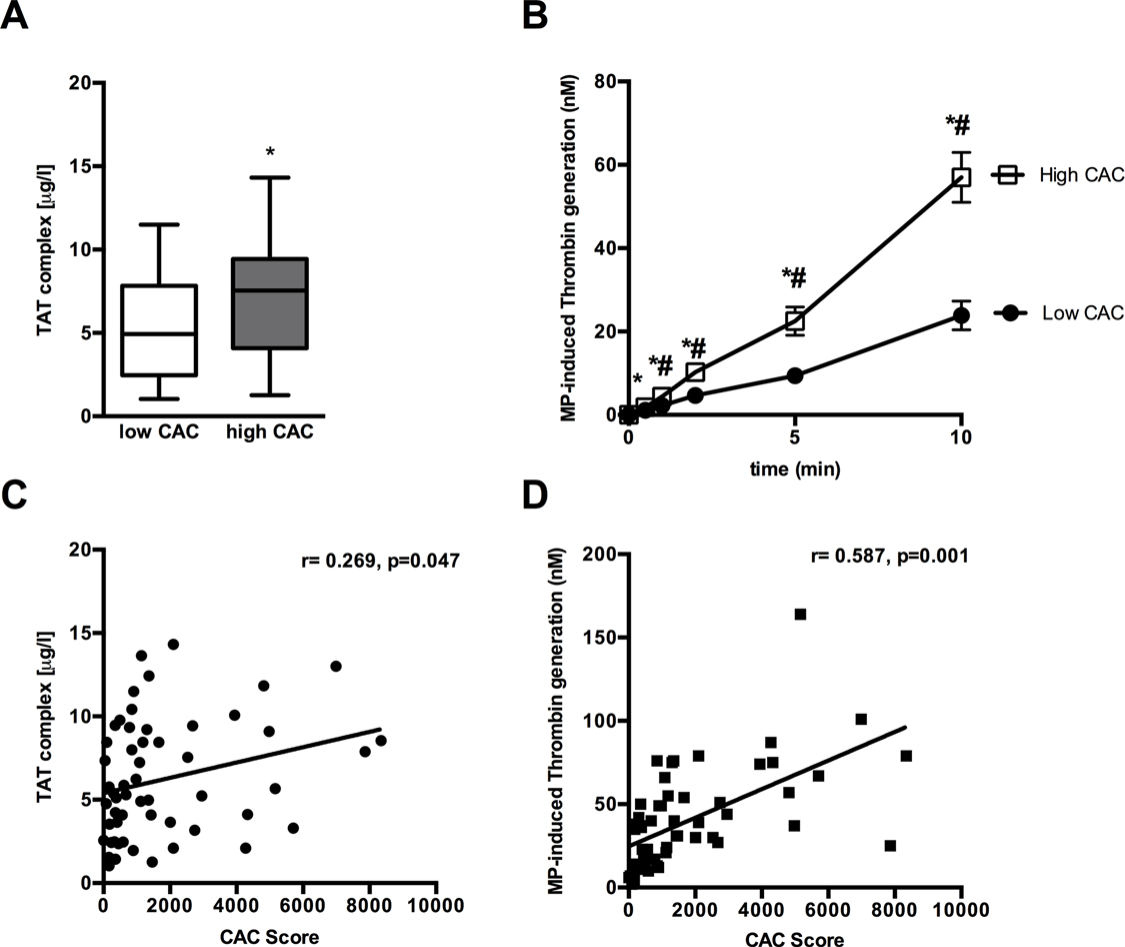
Enhanced plasma thrombin generation and MP-induced thrombin generation in patients with high CAC. (A) In patients with high CAC compared to patients with low CAC TATc level as a marker of thrombin generation in plasma were higher. (C) TATc level correlated with CAC Score. (B) MP-induced thrombin generation was higher in patients with *high* CAC compared to patients with *low* CAC. The kinetic of thrombin generation was assessed by measuring its specific activity on the thrombin substrate at different time points (0–10 min). Data are shown as MW±SE (D) MP-induced thrombin generation correlated with CAC Score. * indicates significant difference of the mean.

In patients with *high* CAC compared to patients with low CAC higher TATc levels as a marker of thrombin generation capacity in plasma were measured (7.2±3.8 μg/l. vs 5.2±3.0 μg/l, p = 0.033) (Fig 2A). Plasma TATc levels correlated positively with CAC Score (r = 0.269, p = 0.047) (Fig 2C).

MP-induced thrombin generation was higher in patients with *high* CAC (57±31 nM after 10 min reaction time) compared to patients with *low* CAC (24±18 nM after 10 min reaction time, p<0.001) (Fig 2B). MP-induced thrombin generation correlated positively with CAC Score (r = 0.587, p = 0.001) (Fig 2D, Table 3). In a multivariate regression analysis, CD41^+^ PMPs, MP-induced thrombin generation and GFR were independent predictors of the severity of CAC in a model including CD62+ EMPs, age, TATc levels and cholesterol (95% CI: 0.061 to 0.328, p = 0.005 and CI: 4.223 to 39.166, p = 0.016 and CI:10.797 to 55.747 respectively) (Table 4).

**Table 4:** Effectors of Coronary artery Calcification by multivariate regression analysis. CD41^+^ PMPs, MP-induced thrombin generation and GFR were independent predictors for the severity of CAC in a model including CD62+ EMPs, age and cholesterol.

	CAC Score	
	B	p Value
CD41^+^ PMPs	0.194	0.005
MP-induced Thrombin generation	22	0.016
CD62E^+^ EMPs	0.309	0.243
Age	-21	0.511
Cholesterol	3.1	0.538
GFR	33	0.005
TAT complex	5.383	0.935
Adjusted R^2^	0.604	

Level of PMPs (r = 0.461, p = 0.001) and CD62E^+^ EMPs (r = 0.300, p = 0.046) correlated positively with MP-induced thrombin generation (Table 5). CD41^+^ PMPs predict MP-induced thrombin generation in a model using CD62E^+^ EMPs, CD31^+^/CD41^-^ EMPs and CD144^+^ EMPs (95% CI: 0.00 to 0.05, p = 0.021) (Table 6).

**Table 5:** Univariate Correlation analysis for the association between MP-induced thrombin generation and endothelial function (FMD) with circulating MPs, CAC Score and AVC Score.

	MP-induced Thrombin generation	
	r	p Value
Coronary Vessel disease (n)	0.554	<0.001
Syntax Score	0.329	<0.001
AVC Score	0.060	0.357
FMD	-0.027	0.709
CD41^+^ PMPs	0.461	<0.001
CD62E^+^ EMPs	0.331	0.001
CD31^+^/CD41^-^ EMPs	0.008	0.920
CD144^+^ EMPs	0.141	0.515
TAT complex	0.283	0.036
	FMD	
	r	
Coronary Vessel disease (n)	-0.059	p Value
Syntax Score	0.094	0.789
AVC Score	-0.065	0.335
CD41^+^ PMPs	-0.006	0.668
CD62E^+^ EMPs	-0.380	0.698
CD31^+^/CD41^-^ EMPs	-0.155	0.578
CD144^+^ EMPs	-0.145	0.533
Thrombin generation	-0.027	0.203

**Table 6:** Effectors of MP-induced thrombin generation by multivariate regression analysis. CD41^+^ PMPs predict MP-induced thrombin generation in a model using CD62E^+^ EMPs, CD31^+^/CD41^-^ EMPs and CD144^+^ EMPs.

	Thrombin generation	
	B	p Value
CD41^+^ PMPs	0.030	0.021
CD62^+^ EMPs	0.009	0.088
CD31^+^/CD41^-^ EMPs	0.011	0.697
CD144+ EMPs	-0.10	0.420
Adjusted R^2^	0.158	

### CAC, MPs and aortic valvular calcification

**Fig 3:**
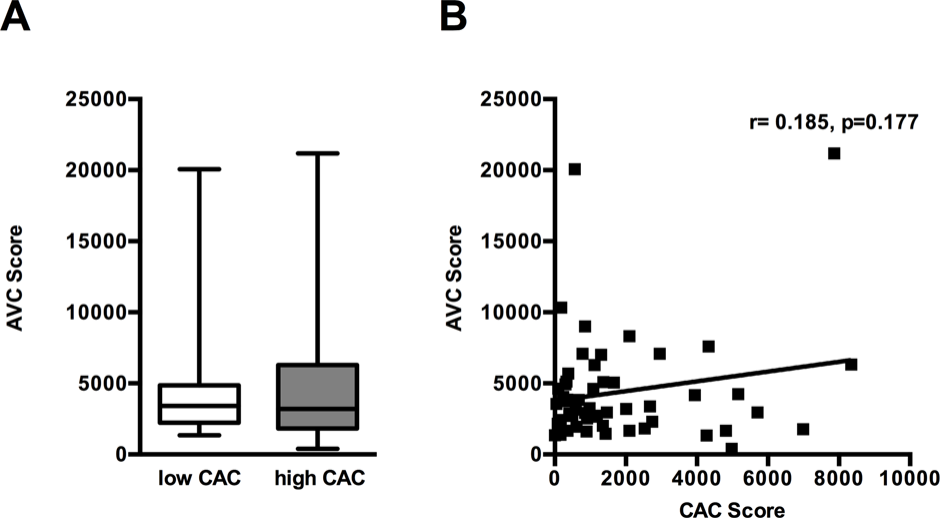
Aortic Valve Calcification (AVC) was not associated with Coronary Artery Calcification (CAC). (A) AVC did not differ between patients with *high* CAC compared to patients with *low* CAC. Data are presented as minimum and maximum (crosses), interquartile range from 25 to 75% (box), mean (square), and median (line) in a box plot. (B) AVC did not correlate with CAC.

AVC Score did not differ between patients with *high* and *low* CAC (4417±3994 vs. 4353±3755, p = 0.9518) (Fig 3). AVC Score did neither correlate with CAC Score (r = -0.06, p = 0.646) (Fig 3, Table 3) nor with TATc levels, MP levels or MP-induced thrombin generation (Table 3).

## Discussion

We here show that in patients with severe aortic valve stenosis MP-induced thrombin generation was independently associated with the severity of CAC but not AVC. The severity of vascular calcification and the clinical burden of coronary artery disease were not associated with severity of valve calcification.

### Coronary atherosclerosis and level of circulating MP

Atherosclerotic lesions frequently calcify as the diseases progresses. The extent of calcification is thought to reflect the total coronary atherosclerotic burden, and can be easily quantified and expressed as coronary artery calcification (CAC) score [21, 22]. CAC is a surrogate measure of advanced coronary atherosclerosis and risk of cardiovascular events [21, 22]. In fact, in our study patients with high CAC presented with higher number of coronary vessels affected and more complex lesions assessed by angiography. However, whereas in acute coronary syndrome early and intermediate stages of calcification may enhance plaque vulnerability, extensive calcification might protect the vessel from rupture by stabilizing the plaque [21, 22]. Thus, CAC might be indicative of a vulnerable patient rather than a vulnerable plaque.

It was previously shown that levels of circulating MPs are elevated in patients with *presence* of atherosclerotic CAD as compared to healthy subjects [4, 6, 7, 23]. Regarding the association between MPs and the *severity* of atherosclerotic processes, Bernard *et al*. measured increased levels of CD144^+^ EMPs in type 2 diabetic patients with coronary non-calcified plaques compared to patients with absence of non-calcified plaques [11]. A correlation between the severity of CAC and CD62E^+^ EMP was shown in postmenopausal woman [12]. We demonstrate for the first time that in patients with advanced atherosclerotic disease and aortic valve stenosis levels of PMPs as well as CD62E^+^ EMPs are independently associated with the severity of CAC. As a limitation, the role of leucocytes-derived MPs as well as erythrocyte-derived MPs has still to been investigated, both subpopulation of MPs which originate from activated monocytes and atherosclerotic plaques respectively red blood cells and which potentially contribute to thrombin generation [24], atherosclerotic inflammation and plaque progression [25].

### Coronary atherosclerosis, plasma thrombin generation and MP-induced thrombin generation

Thrombin is generated in plasma by cleavage of prothrombin into thrombin triggered by tissue factor (TF) or phosphatidylserine on platelets, endothelial cells, atherosclerotic macrophages and smooth muscle cells [26]. Generated at sites of vascular injury in the vicinity of a thrombus, thrombin promotes platelet activation, adhesion, and trafficking of inflammatory cells into sites of injury [15, 16]. Moreover, thrombin exerts pleiotropic effects on the endothelium controlling the proliferative/reparative responses to injury and promoting both proinflammatory and procoagulant endothelial phenotypes [15, 16]. In animal studies, alterations in clotting activity or direct thrombin inhibition attenuate the progression and thrombogenicity of atherosclerotic plaques [15, 27]. Here, we show that higher thrombin generation in human plasma (as assessed by TATc level) were associated with higher CAC scores. Our data are in line with recently published work of Borissoff et al demonstrating in patients with suspected CAD that plasma thrombin generation is independently associated with the CAC Score [17].

In the plasma, a major source of phosphatidylserine and tissue factor are circulating MPs. The expression of these markers on the surface of MPs determines the procoagulant activity and MP-induced thrombin generation [13, 14]. In patients with moderate CAD Del Turco et al. have shown an association between MPs procoagulant activity and atherosclerotic plaque morphology but did not measure MP level in their study [28]. We here show that thrombin generation of circulating MPs is independently associated with severity of CAC. As thrombin plays a role in the pathophysiology of atherosclerosis progression [15, 16] our data indicate that MPs are not only a result of atherosclerotic modifications affecting the vascular compartment but also might contribute to the progression of coronary atherosclerosis. The progression of atherosclerosis leads to the release of new MPs into the circulation potentially creating a vicious cycle.

We were not able to distinguish between the subpopulations of MPs by means of their contribution to thrombin generation as thrombin generation of total pool of plasma MPs was measured. Phosphatidylserine promotes the binding of the coagulation factors, assembly a prothrombinase complex to cleave prothrombin into thrombin. TF is a receptor for VII/VIIa. The TF:FVIIIa complex activates both FX and FIX to initiate blood coagulation [13]. Studies using *in vitro* generated MPs demonstrated that the mechanism to promote thrombin might differ between the various MP phenotypes. PMPs, EMPs and RBC-MPs have strong procoagulant properties due to exposure of phopshatidylserine. However, PMPs and erythrocyte-derived MPs can also initiate thrombin generation independently of TF in a FII-dependent manner [24]. As the majority of TF expressing MPs seems to derive from monocytes this might be the major mechanism of this MP phenotype to promote thrombin generation [13].

Until now, data about the potential mechanism of thrombin generation of different MP subpopulation were obtained by generation of MP subpopulation by *in vitro* preperation and stimulation of separated blood cell populations or cell culture to generate MPs [13, 24]. These isolated MPs subpopulation are used to assess MP subpopulation induced thrombin generation. Molecular mechanisms of *in vitro* generated isolated MP subpopulation may be different from those triggered by MPs generated *in vivo*. Using the circulating pool of total MPs of patients as in our study may even be more accurate to quantify the thrombin generation of circulating MPs *in vivo*.

In our study, only PMPs predict MP-induced thrombin generation and severity of CAC suggesting that level of EMPs might be much more the result of atherosclerosis and the disturbed endothelial integrity [4, 29]. In fact, along with previous studies [30, 31] in our study level of EMPs correlate with endothelial dysfunction measured by FMD. Therefore, EMPs can be viewed as circulating markers of a compromised endothelial integrity [32]. Taken together, we suggest that the PMP-induced but not EMP-induced thrombin generation play a role in the progress of coronary atherosclerosis.

MPs, including EMPs may promote atherosclerosis progression also independently of thrombin generation at various stages [4, 33]. Circulating MPs can attenuate endothelium-dependent relaxation and impair the atheroprotective function of the vascular endothelium by inhibition of the nitric oxide pathway [34, 35] as shown *ex vivo*. *In vitro* and *in vivo* generated MPs promote inflammation within the vascular wall by promoting leucocyte adhesion and migration towards atherosclerotic lesions [4, 36]. MPs contain microRNA that are thought to be involved in controlling inflammation in atherosclerotic disease [8, 37]. In advanced lesions, plaque-derived MPs stimulate *in vivo* neovascularization, likely leading to proliferation and plaque vulnerability and contribute to the transition into unstable lesions [4, 38]. At the time of rupture, highly thrombogenic MPs are released locally further increasing local thrombus formation [4, 39] and might contribute to onset of acute coronary syndrome [40, 41]. Taken together, circulating MPs might be involved in the progression of atherosclerosis by MP-induced thrombin generation as well as thrombin-independent effects on vascular homeostasis.

### Coronary atherosclerosis and aortic valve calcification

Aortic valve stenosis is independently associated with traditional cardiovascular risk factors and clinically apparent cardiovascular disease [2], so that some authors claim that the degeneration of the aortic valve may represent an atherosclerosis-like process involving both the aortic valve as well as the vascular system [3]. However, in our study, we found no association between AVC score and CAC score, MPs level or MP-induced thrombin generation. In line with our results, other studies also showed that AVC Score was not associated with CAC Score [42, 43]. Recently, Henein et al. demonstrated that aortic root calcification and not valve calcification correlates with CAC [44]. In several prospective trials therapeutic approaches preventing the progression of vascular atherosclerosis (e.g. lipid-lowering therapy with statins) failed to prevent progression of valve sclerosis [45, 46]. Taken together, our study underscores distinct pathophysiological mechanisms of atherosclerosis and aortic valve stenosis.

Our study has several limitations. This was an investigation at a single time point. Hence the association between circulating MPs and the induction and further progression of atherosclerosis remains unclear. MPs with a size between 0.1–0.2 μm were not accounted for in this way. Thus, the number of MPs determined by flow cytometry was an underestimation of the true count. Further studies are required to investigate the mechanistic link between MP-induced thrombin generation and atherosclerosis.

## Conclusion

In patients with severe aortic valve stenosis MP-induced thrombin generation was independently associated with the severity of CAC but not AVC indicating different pathomechanism involved in coronary artery and aortic valve calcification.

## Supporting Information

S1 Fig Representative flow cytometry plots of circulating MPs. (A) Gating population of MPs by using microbead standard of 1.0 μm as upper limit. (B-D) Discriminating MP subpopulation according to the expression of established surface antigens and matching isotype controls. Platelet-derived MPs (PMPs) were defined as CD41^+^-MPs (B). EMP subpopulations were defined as CD31^+^/CD41^-^ (B), CD144^+^ (C) or CD62E^+^ (D).
(TIFF)

S1 File Underlying data of the n = 55 patients. Coronary Calcification Score (CAC), Aortic Valve Calcification Score (AVC), Level of Platelets derived MPs (CD41+ Mps), level of endothelial derived MPs (CD62E+, CD144+ and CD31+/Cd41- MPs), MP induced thrombin generation (MP-thrombin), flow mediated dilation (FMD) and level of Thrombin-antithrombin complex (TATc).
(PDF)

## References

[1] Hansson GK. Inflammation, atherosclerosis, and coronary artery disease. N Engl J Med. 2005;352(16):1685–95. Epub 2005/04/22. 10.1056/NEJMra043430 . 15843671

[2] Otto CM, Lind BK, Kitzman DW, Gersh BJ, Siscovick DS. Association of aortic-valve sclerosis with cardiovascular mortality and morbidity in the elderly. N Engl J Med. 1999;341(3):142–7. 10.1056/NEJM199907153410302 . 10403851

[3] Wierzbicki A, Shetty C. Aortic stenosis: an atherosclerotic disease? J Heart Valve Dis. 1999;8(4):416–23. . 10461242

[4] Rautou PE, Vion AC, Amabile N, Chironi G, Simon A, Tedgui A, et al Microparticles, vascular function, and atherothrombosis. Circ Res. 2011;109(5):593–606. 10.1161/CIRCRESAHA.110.233163 . 21852557

[5] Schiro A, Wilkinson FL, Weston R, Smyth JV, Serracino-Inglott F, Alexander MY. Endothelial microparticles as conveyors of information in atherosclerotic disease. Atherosclerosis. 2014;234(2):295–302. Epub 2014/04/12. 10.1016/j.atherosclerosis.2014.03.019 . 24721189

[6] Sinning JM, Losch J, Walenta K, Bohm M, Nickenig G, Werner N. Circulating CD31+/Annexin V+ microparticles correlate with cardiovascular outcomes. Eur Heart J. 2011;32(16):2034–41. 10.1093/eurheartj/ehq478 . 21186238

[7] Amabile N, Cheng S, Renard JM, Larson MG, Ghorbani A, McCabe E, et al Association of circulating endothelial microparticles with cardiometabolic risk factors in the Framingham Heart Study. Eur Heart J. 2014 Epub 2014/04/20. 10.1093/eurheartj/ehu153 . 24742886 PMC4223610

[8] Jansen F, Yang X, Proebsting S, Hoelscher M, Przybilla D, Baumann K, et al MicroRNA expression in circulating microvesicles predicts cardiovascular events in patients with coronary artery disease. Journal of the American Heart Association. 2014;3(6):e001249 Epub 2014/10/29. 10.1161/JAHA.114.001249 25349183 PMC4338711

[9] Diehl P, Nagy F, Sossong V, Helbing T, Beyersdorf F, Olschewski M, et al Increased levels of circulating microparticles in patients with severe aortic valve stenosis. Thromb Haemost. 2008;99(4):711–9. 10.1160/TH07-05-0334 . 18392329

[10] Horn P, Stern D, Veulemans V, Heiss C, Zeus T, Merx MW, et al Improved endothelial function and decreased levels of endothelium-derived microparticles after transcatheter aortic valve implantation. EuroIntervention: journal of EuroPCR in collaboration with the Working Group on Interventional Cardiology of the European Society of Cardiology. 2015;10(12):1456–63. Epub 2014/10/08. 10.4244/EIJY14M10_02 . 25287265

[11] Bernard S, Loffroy R, Serusclat A, Boussel L, Bonnefoy E, Thevenon C, et al Increased levels of endothelial microparticles CD144 (VE-Cadherin) positives in type 2 diabetic patients with coronary noncalcified plaques evaluated by multidetector computed tomography (MDCT). Atherosclerosis. 2009;203(2):429–35. 10.1016/j.atherosclerosis.2008.07.039 . 18804209

[12] Jayachandran M, Litwiller RD, Owen WG, Heit JA, Behrenbeck T, Mulvagh SL, et al Characterization of blood borne microparticles as markers of premature coronary calcification in newly menopausal women. Am J Physiol Heart Circ Physiol. 2008;295(3):H931–H8. Epub 2008/07/16. 00193.2008 [pii] 10.1152/ajpheart.00193.2008 18621859 PMC2544500

[13] Owens AP 3rd, Mackman N. Microparticles in hemostasis and thrombosis. Circ Res. 2011;108(10):1284–97. 10.1161/CIRCRESAHA.110.233056 21566224 PMC3144708

[14] Lacroix R, Dignat-George F. Microparticles as a circulating source of procoagulant and fibrinolytic activities in the circulation. Thromb Res. 2012;129 Suppl 2:S27–9. 10.1016/j.thromres.2012.02.025 . 22424856

[15] Borissoff JI, Spronk HM, ten Cate H. The hemostatic system as a modulator of atherosclerosis. N Engl J Med. 2011;364(18):1746–60. Epub 2011/05/06. 10.1056/NEJMra1011670 . 21542745

[16] Kalz J, ten Cate H, Spronk HM. Thrombin generation and atherosclerosis. J Thromb Thrombolysis. 2014;37(1):45–55. Epub 2013/11/19. 10.1007/s11239-013-1026-5 . 24241912

[17] Borissoff JI, Joosen IA, Versteylen MO, Spronk HM, ten Cate H, Hofstra L. Accelerated in vivo thrombin formation independently predicts the presence and severity of CT angiographic coronary atherosclerosis. JACC Cardiovascular imaging. 2012;5(12):1201–10. Epub 2012/12/15. 10.1016/j.jcmg.2012.01.023 . 23236969

[18] Dimitrow PP, Hlawaty M, Undas A, Sniezek-Maciejewska M, Sobien B, Stepien E, et al Effect of aortic valve stenosis on haemostasis is independent from vascular atherosclerotic burden. Atherosclerosis. 2009;204(2):e103–8. Epub 2009/01/28. 10.1016/j.atherosclerosis.2008.12.029 . 19171341

[19] Horn P, Cortese-Krott MM, Amabile N, Hundsdorfer C, Kroncke KD, Kelm M, et al Circulating microparticles carry a functional endothelial nitric oxide synthase that is decreased in patients with endothelial dysfunction. Journal of the American Heart Association. 2012;2(1):e003764 10.1161/JAHA.112.003764 23525410 PMC3603231

[20] Erdbrugger U, Rudy CK, M EE, Dryden KA, Yeager M, Klibanov AL, et al Imaging flow cytometry elucidates limitations of microparticle analysis by conventional flow cytometry. Cytometry A. 2014;85(9):756–70. Epub 2014/06/07. 10.1002/cyto.a.22494 . 24903900

[21] Alexopoulos N, Raggi P. Calcification in atherosclerosis. Nature reviews Cardiology. 2009;6(11):681–8. Epub 2009/09/30. 10.1038/nrcardio.2009.165 . 19786983

[22] Nicoll R, Henein MY. Arterial calcification: friend or foe? Int J Cardiol. 2013;167(2):322–7. Epub 2012/07/20. 10.1016/j.ijcard.2012.06.110 . 22809537

[23] Lee ST, Chu K, Jung KH, Kim JM, Moon HJ, Bahn JJ, et al Circulating CD62E+ microparticles and cardiovascular outcomes. PloS one. 2012;7(4):e35713 10.1371/journal.pone.0035713 22563392 PMC3338519

[24] Van Der Meijden PE, Van Schilfgaarde M, Van Oerle R, Renne T, ten Cate H, Spronk HM. Platelet- and erythrocyte-derived microparticles trigger thrombin generation via factor XIIa. J Thromb Haemost. 2012;10(7):1355–62. 10.1111/j.1538-7836.2012.04758.x . 22537188

[25] Wang JG, Aikawa E, Aikawa M. Leukocyte-derived microparticles as proinflammatory mediators in atherosclerosis. J Am Coll Cardiol. 2013;62(16):1442–5. Epub 2013/05/28. 10.1016/j.jacc.2013.04.054 . 23707315

[26] ten Cate H. Tissue factor-driven thrombin generation and inflammation in atherosclerosis. Thromb Res. 2012;129 Suppl 2:S38–40. Epub 2012/03/09. 10.1016/j.thromres.2012.02.028 . 22398011

[27] Bea F, Kreuzer J, Preusch M, Schaab S, Isermann B, Rosenfeld ME, et al Melagatran reduces advanced atherosclerotic lesion size and may promote plaque stability in apolipoprotein E-deficient mice. Arterioscler Thromb Vasc Biol. 2006;26(12):2787–92. Epub 2006/09/23. 10.1161/01.ATV.0000246797.05781.ad . 16990551

[28] Del Turco S, Basta G, Mazzarisi A, Battaglia D, Navarra T, Coceani M, et al Procoagulant activity of circulating microparticles is associated with the presence of moderate calcified plaque burden detected by multislice computed tomography. Journal of geriatric cardiology: JGC. 2014;11(1):13–9. Epub 2014/04/22. 10.3969/j.issn.1671-5411.2014.01.008 24748876 PMC3981978

[29] Dignat-George F, Boulanger CM. The many faces of endothelial microparticles. Arterioscler Thromb Vasc Biol. 2011;31(1):27–33. 10.1161/ATVBAHA.110.218123 . 21160065

[30] Werner N, Wassmann S, Ahlers P, Kosiol S, Nickenig G. Circulating CD31+/annexin V+ apoptotic microparticles correlate with coronary endothelial function in patients with coronary artery disease. Arterioscler Thromb Vasc Biol. 2006;26(1):112–6. Epub 2005/10/22. 01.ATV.0000191634.13057.15 [pii] 10.1161/01.ATV.0000191634.13057.15 . 16239600

[31] Horn P, Amabile N, Angeli FS, Sansone R, Stegemann B, Kelm M, et al Dietary flavanol intervention lowers the levels of endothelial microparticles in coronary artery disease patients. Br J Nutr. 2014;111(7):1245–52. Epub 2013/11/30. 10.1017/S0007114513003693 . 24286443

[32] Sabatier F, Camoin-Jau L, Anfosso F, Sampol J, Dignat-George F. Circulating endothelial cells, microparticles and progenitors: key players towards the definition of vascular competence. J Cell Mol Med. 2009;13(3):454–71. 10.1111/j.1582-4934.2008.00639.x . 19379144 PMC3822508

[33] Tushuizen ME, Diamant M, Sturk A, Nieuwland R. Cell-derived microparticles in the pathogenesis of cardiovascular disease: friend or foe? Arterioscler Thromb Vasc Biol. 2011;31(1):4–9. 10.1161/ATVBAHA.109.200998 . 21160062

[34] Boulanger CM, Scoazec A, Ebrahimian T, Henry P, Mathieu E, Tedgui A, et al Circulating microparticles from patients with myocardial infarction cause endothelial dysfunction. Circulation. 2001;104(22):2649–52. Epub 2001/11/28. . 11723013 10.1161/hc4701.100516

[35] Brodsky SV, Zhang F, Nasjletti A, Goligorsky MS. Endothelium-derived microparticles impair endothelial function in vitro. Am J Physiol Heart Circ Physiol. 2004;286(5):H1910–5. Epub 2004/04/10. 10.1152/ajpheart.01172.2003 286/5/H1910 [pii]. . 15072974

[36] Rautou PE, Leroyer AS, Ramkhelawon B, Devue C, Duflaut D, Vion AC, et al Microparticles from human atherosclerotic plaques promote endothelial ICAM-1-dependent monocyte adhesion and transendothelial migration. Circ Res. 2011;108(3):335–43. 10.1161/CIRCRESAHA.110.237420 . 21164106

[37] Hulsmans M, Holvoet P. MicroRNA-containing microvesicles regulating inflammation in association with atherosclerotic disease. Cardiovasc Res. 2013;100(1):7–18. Epub 2013/06/19. 10.1093/cvr/cvt161 . 23774505

[38] Leroyer AS, Rautou PE, Silvestre JS, Castier Y, Leseche G, Devue C, et al CD40 ligand+ microparticles from human atherosclerotic plaques stimulate endothelial proliferation and angiogenesis a potential mechanism for intraplaque neovascularization. J Am Coll Cardiol. 2008;52(16):1302–11. Epub 2008/10/22. 10.1016/j.jacc.2008.07.032 . 18929241

[39] Leroyer AS, Isobe H, Leseche G, Castier Y, Wassef M, Mallat Z, et al Cellular origins and thrombogenic activity of microparticles isolated from human atherosclerotic plaques. J Am Coll Cardiol. 2007;49(7):772–7. 10.1016/j.jacc.2006.10.053 . 17306706

[40] Morel O, Pereira B, Averous G, Faure A, Jesel L, Germain P, et al Increased levels of procoagulant tissue factor-bearing microparticles within the occluded coronary artery of patients with ST-segment elevation myocardial infarction: role of endothelial damage and leukocyte activation. Atherosclerosis. 2009;204(2):636–41. 10.1016/j.atherosclerosis.2008.10.039 . 19091315

[41] Porto I, Biasucci LM, De Maria GL, Leone AM, Niccoli G, Burzotta F, et al Intracoronary microparticles and microvascular obstruction in patients with ST elevation myocardial infarction undergoing primary percutaneous intervention. Eur Heart J. 2012;33(23):2928–38. 10.1093/eurheartj/ehs065 . 22453653

[42] Mohler ER 3rd, Medenilla E, Wang H, Scott C. Aortic valve calcium content does not predict aortic valve area. J Heart Valve Dis. 2006;15(3):322–8. Epub 2006/06/21. . 16784067

[43] Koos R, Mahnken AH, Sinha AM, Wildberger JE, Hoffmann R, Kuhl HP. Aortic valve calcification as a marker for aortic stenosis severity: assessment on 16-MDCT. AJR American journal of roentgenology. 2004;183(6):1813–8. Epub 2004/11/18. 10.2214/ajr.183.6.01831813 . 15547235

[44] Henein M, Hallgren P, Holmgren A, Sorensen K, Ibrahimi P, Kofoed KF, et al Aortic root, not valve, calcification correlates with coronary artery calcification in patients with severe aortic stenosis: A two-center study. Atherosclerosis. 2015;243(2):631–7. Epub 2015/11/10. 10.1016/j.atherosclerosis.2015.10.014 . 26551591

[45] Rossebo AB, Pedersen TR, Boman K, Brudi P, Chambers JB, Egstrup K, et al Intensive lipid lowering with simvastatin and ezetimibe in aortic stenosis. N Engl J Med. 2008;359(13):1343–56. Epub 2008/09/04. 10.1056/NEJMoa0804602 . 18765433

[46] Chan KL, Teo K, Dumesnil JG, Ni A, Tam J, Investigators A. Effect of Lipid lowering with rosuvastatin on progression of aortic stenosis: results of the aortic stenosis progression observation: measuring effects of rosuvastatin (ASTRONOMER) trial. Circulation. 2010;121(2):306–14. Epub 2010/01/06. 10.1161/CIRCULATIONAHA.109.900027 . 20048204

