# Supplementary figures and images for: Microparticle-Induced Coagulation Relates to Coronary Artery Atherosclerosis in Severe Aortic Valve Stenosis
Source: PLoS One. 2016 Mar 24;11(3):e0151499. doi: 10.1371/journal.pone.0151499 (PMC4807100; doi:10.1371/journal.pone.0151499)

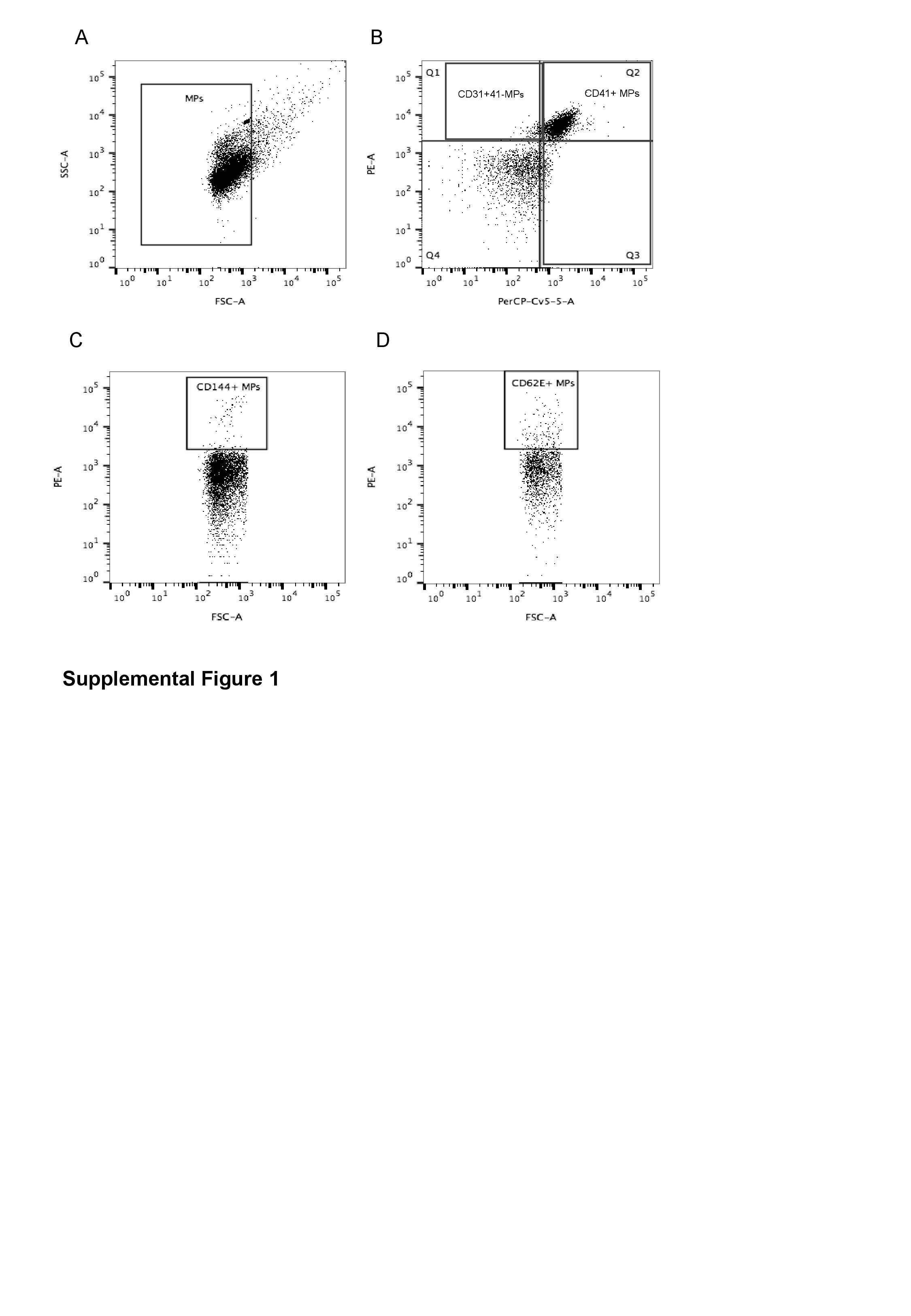

Supplement: S1 Fig — (A) Gating population of MPs by using microbead standard of 1.0 μm as upper limit. (B-D) Discriminating MP subpopulation according to the expression of established surface antigens and matching isotype controls. Platelet-derived MPs (PMPs) were defined as CD41+-MPs (B). EMP subpopulations were defined as CD31+/CD41- (B), CD144+ (C) or CD62E+ (D). (TIFF) [file pone.0151499.s001.tiff]
