# Supplementary material for: Microparticle-Induced Coagulation Relates to Coronary Artery Atherosclerosis in Severe Aortic Valve Stenosis
Source: PLoS One. 2016 Mar 24;11(3):e0151499. doi: 10.1371/journal.pone.0151499 (PMC4807100; doi:10.1371/journal.pone.0151499)
Supplement: S1 File — Coronary Calcification Score (CAC), Aortic Valve Calcification Score (AVC), Level of Platelets derived MPs (CD41+ Mps), level of endothelial derived MPs (CD62E+, CD144+ and CD31+/Cd41- MPs), MP induced thrombin generation (MP-thrombin), flow mediated dilation (FMD) and level of Thrombin-antithrombin complex (TATc). (PDF) [file pone.0151499.s002.pdf]

CAC	AVC	CD41+ MPs	CD62E+ MPs	CD144+ MPs	CD31+/41- Mps	MP-Thrombin	FMD	TATc level
1	1357	749	623	185	230	6	2,3	2,6
47	3562	432	912	320	133	7	2,8	7,3
83	2157	724	1603	1118	66	6	3,2	4,8
96	4626	544	255	280	300	2	2,9	8,5
156	1389	565	1252	261	243	14	2,5	1,5
165	2424	490	323	198	421	9	2,5	1,0
167	2154	505	201	263	356	4	3,5	5,8
180	10326	862	503	230	304	35	2,9	3,5
237	4043	203	1122	171	220	38	2,9	2,4
299	4928	755	377	180	520	42	2,4	5,4
328	5132	1229	300	800	250	11	3,5	2,5
350	3856	681	422	579	136	37	2,7	9,5
355	3744	651	145	139	434	13	3	1,4
358	1668	187	420	222	147	50	2,9	4,2
379	5698	619	222	395	399	36	3,6	5,1
405	2884	773	259	219	73	23	3,8	3,7
450	3807	731	612	182	290	17	3,2	2,4
494	2711	1124	494	248	225	12	3,3	9,8
563	20064	680	1507	1280	340	23	2,6	4,1
587	3141	876	423	860	247	10	3	2,5
613	1957	2640	746	190	338	17	3	5,9
673	3841	664	2912	435	112	40	2	5,3
780	7089	1055	2876	530	307	17	1,8	9,3
848	2920	7040	1624	174	160	13	2,2	8,0
851	9004	1450	832	108	170	76	3,9	10,4
896	1617	7474	1330	350	190	12	2,7	2,0
904	2534	5005	2424	252	88	49	3,4	11,5
975	3263	843	612	230	89	49	3,4	6,2
1084	4620	9551	1928	120	115	66	3,1	7,2
1121	6293	1950	853	347	189	21	3,2	4,9

1139	2730	3965	2471	277	313	24	3,2	13,6
1184	2662	1028	1181	148	725	55	2,8	8,5
1305	7018	4069	1932	1330	437	75	2,7	9,2
1349	2021	3918	1911	739	291	76	3,6	5,0
1367	5085	2671	721	136	188	40	2,8	12,4
1426	1447	500	1063	288	145	31	3,5	4,1
1462	2957	2140	1982	831	300	31	3,5	1,3
1660	5046	8985	352	104	352	54	3,2	8,5
2011	3197	2702	1545	106	135	30	2,4	3,7
2098	8317	777	501	337	249	79	3,3	14,3
2099	1662	3572	624	450	116	39	3,3	2,1
2529	1826	3308	1709	105	302	30	2,5	7,5
2675	3379	665	1746	107	154	27	3	9,4
2741	2296	1721	480	334	152	51	4,2	3,2
2951	7075	810	2192	240	236	44	2,1	5,2
3941	4166	1450	2000	142	410	74	2,6	10,1
4272	1328	1600	1912	373	131	87	2,8	2,1
4333	7603	1328	1675	183	103	75	2,7	4,1
4816	1670	13500	669	289	450	57	4,5	11,8
4978	402	4171	2421	455	262	37	2,9	9,1
5160	4237	9250	852	250	392	164	2,5	5,7
5704	2947	4548	2535	158	200	67	2,4	3,3
6988	1766	13500	1271	405	270	101	2	13,0
7865	21182	4554	563	338	195	25	3,3	7,9
8346	6323	13414	899	141	354	79	3	8,6

Underlying data of the n=55 patients. Coronary Calcification Score **(CAC)**, Aortic Valve Calcification Score (**AVC**), Level of Platelets derived MPs (**CD41+ Mps**), level of endothelial derived MPs (**CD62E+, CD144+ and CD31+/Cd41- MPs**), MP induced thrombin generation **(MP-thrombin)**, flow mediated dilation (**FMD**) and level of Thrombin-antithrombin complex (**TATc**).
